# We should learn from the Asia–Pacific responses to COVID-19

**DOI:** 10.1016/j.lanwpc.2020.100062

**Published:** 2020-11-27

**Authors:** Jay Patel, Devi Sridhar

**Affiliations:** a Global Health Governance Programme, Usher Institute of Population Health Sciences and Informatics, University of Edinburgh, Edinburgh, UK; b Faculty of Medicine and Health, University of Leeds, Leeds, UK; c College of Medicine and Veterinary Medicine, University of Edinburgh, Edinburgh, UK

**Keywords:** COVID-19, Epidemiology, Health policy, Public health

The relative global successes of strategies to control the COVID-19 pandemic, keep their economies afloat and avoid longer, harsh lockdown measures are markedly skewed towards the Asia–Pacific region. These successes are largely attributable to the urgent action taken by many Asia–Pacific jurisdictions to eliminate community transmission through a series of non-pharmaceutical interventions: a ‘zero-COVID’ strategy. In parts of western Europe and the Americas, premature easing of restrictions has preceded resurgences of SARS-CoV-2 infections. These countries are confronted with the challenge of devising new strategies that give precedence to the health of citizens, whilst minimising further economic damage, often in climates of reduced public compliance and government mistrust. The danger of reimposing restrictions on population movement, in the absence of policies that enable a zero-COVID strategy, are unsustainable lockdown–and–release cycles that compound the pervasive damage created by this syndemic. Aspiring towards the approaches observed in Taiwan and New Zealand, can considerably mitigate further devastation, at least until science permits a safe and effective vaccine.

Given the marked international variance in epidemic responses, comparative analyses of the different approaches and experiences offer the opportunity for cross-country learning and facilitate important lessons to be shared. In *The Lancet Regional Health – Western Pacific*, Jennifer Summers and colleagues examine the public health approaches employed by Taiwan and New Zealand, up to August 2020, and offer six recommendations for other high-income countries [1]. The authors performed literature searches across scientific databases, preprint repositories, and government websites, including a total of 32 articles in their review. Their evaluation adds strength to the argument for prioritising the elimination of domestic transmission, to ensure maximum protection of both public health and the economy.

Despite contrasting pre-pandemic preparedness and initial responses, both countries observed relatively few cases and low mortality up to August 2020: New Zealand (1 934 cases and 25 deaths), and Taiwan (550 cases and 7 deaths). The latest World Economic Outlook report––produced by the International Monetary Fund––reveal that up to October 2020, New Zealand experienced an annual contraction in real gross domestic product (GDP) of 6.1%, whereas Taiwan sustained a net GDP change of zero [2]. Despite considerable economic decline in New Zealand, this is significantly lower than countries across western Europe and the Americas, where national disease control fared less successful, for instance: Spain (−12.8%), Argentina (−11.8%), Italy (−10.6%), France (−9.8%), and the United Kingdom (−9.8%) [2].

The responses in each nation (Table 1) demonstrate how rapid and systematic interventions prioritising testing, effective contact tracing and isolation; strict border control; and clear public health messaging can substantially curb viral transmission. The Taiwanese approach in particular, exemplifies that elimination or suppression does not have to mean lockdown. These are three clear steps that all countries can take to sustainably suppress SARS-CoV-2.

**Table 1:** Comparison of approaches, response measures, and interventions in Taiwan and New Zealand up to August 2020.

Country	Overall strategy	Knowledge of infection status	Community engagement	Public-health capacity	Health-system capacity	Measures for border control
New Zealand	Four-level alert system	No publicly specified indicator	Social bubble approach allowed gradual expansion of small and exclusive social groups; no physical distancing required at alert level one; mask wearing on public transport mandated in August	Testing capacity increased from 1 500 PCR tests per day in March to 3 700 tests per day in April with plans to reach 5 000 capacity; manual and app-based tracing being done	Some expansion of ICU capacity and number of staff trained to use ICU equipment; ICU bed capacity of 358 (71.6 ICU beds per million) and ventilator capacity of 334 (66.8 million ventilators per million)	Border closed to most visitors; all arrivals are tested and quarantined for 14 days
Taiwan	Three-level approach	No publicly specified indicator	1.5 m physical distancing in indoor settings, reduced to 1 m when outdoors and face mask required in high-risk settings	Daily PCR testing capacity increased from 1 300 in February to 7 166 per day in July; manual and electronic tracing systems in use	Real-time monitoring of ICU capacity, ventilators and patient ward numbers by hospital maintained and continually monitored; ICU bed capacity of 7 090 (308.3 ICU beds per million) and ventilator capacity of 9 932 (431.8 million ventilators per million)	All arrivals must submit a health declaration form, capturing details of travel history and any disease symptoms, testing, and 14-day home quarantine

Data have been adapted from the comparative framework for COVID-19 lockdown exit strategies developed by Han and colleagues [3]. A detailed comparison of the specific response measures and interventions in both countries are available in the appendix of the original review. ICU=intensive care unit. PCR=polymerase chain reaction.

Modelling studies suggest population-based interventions were instrumental in Taiwan's initial elimination efforts, and case-based interventions alone were not sufficient control measures [4]. Given the additional factor of public fatigue, adherence to restrictions is presented with new challenges that must be addressed to ensure maximum voluntary compliance. Six recommendations to guide the current and inevitable future pandemics conclude this review, synthesising the possible defining features of the successful responses: (1) establish or strengthen a national agency dedicated to preventing and controlling public health threats; (2) formulate a non-specific pandemic plan; (3) invest in resources and infrastructure for future disease threats; (4) enhance training programmes pertaining to effective pandemic management and public health development; (5) develop systems for evaluating pandemic responses; and (6) create wider acceptability for pandemic response measures.

Given the limitations of this analysis, caution is merited in the extent to which recommendations can be extrapolated to other nations. The evaluation did not identify direct associations between the specific interventions and their relative effect on epidemic control. The authors have attributed the combination of these strategic measures towards a successful outcome, but mandating these many not be as easily attainable in nations of different income levels and geographical location. Instituting strict border controls and quarantine facilities may also be viewed as less exacting in island nations. Further population-based epidemiological and modelling studies are needed to ascertain the true, independent effect of specific response measures.

Taiwan were better prepared given the existing public health infrastructure from the SARS outbreak, but other nations also had established surveillance and warning systems to manage infectious disease threats. The proactive leadership style in Taiwan and New Zealand mobilised these impressively, in the context of SARS-CoV-2. There is an ongoing need to develop high-quality scientific data for efficient translation into evidence-based decision-making within the wider public health governance framework, especially if future waves of infection are to emerge.

The Asia–Pacific region models to the rest of the world that a zero-COVID strategy works and should be prioritised as a highly effective strategic response.

## Author Contributions

Jay Patel critically appraised the original review and drafted the manuscript. Devi Sridhar developed the comparative framework table and critically revised the manuscript.

## Declaration of Interests

Professor Sridhar sits on the Scottish Government COVID-19 advisory group, has attended Scientific Advisory Group for Emergencies and UK Cabinet Office Advisory Group meetings, and sits on the Royal Society Data Evaluation and Learning for Viral Epidemics initiative that inputs into the Scientific Advisory Group for Emergencies.
